# *SP110* Polymorphisms Are Genetic Markers for Vulnerability to Latent and Active Tuberculosis Infection in Taiwan

**DOI:** 10.1155/2018/4687380

**Published:** 2018-12-05

**Authors:** So-Yi Chang, Mei-Ling Chen, Meng-Rui Lee, Yun-Chieh Liang, Tzu-Pin Lu, Jann-Yuan Wang, Bo-Shiun Yan

**Affiliations:** ^1^Institute of Biochemistry and Molecular Biology, National Taiwan University Medical College, Taipei 10051, Taiwan; ^2^Graduate Institute of Oncology, National Taiwan University Medical College, Taipei 10051, Taiwan; ^3^Department of Internal Medicine, National Taiwan University Hospital and National Taiwan University Medical College, Taipei 10051, Taiwan; ^4^Clinical Trial Center, National Taiwan University Hospital, Taipei 10055, Taiwan; ^5^Institute of Epidemiology and Preventive Medicine, National Taiwan University College of Public Health, Taipei 10055, Taiwan

## Abstract

One-fourth of the human population is estimated to have been exposed to *Mycobacterium tuberculosis* (*Mtb*) and carries the infection in its latent form. This latent infection presents a lifelong risk of developing active tuberculosis (TB) disease, and persons with latent TB infection (LTBI) are significant contributors to the pool of active TB cases. Genetic polymorphisms among hosts have been shown to contribute to the outcome of *Mtb* infection. The *SP110* gene, which encodes an interferon-induced nuclear protein, has been shown to control host innate immunity to *Mtb* infection. In this study, we provide experimental data demonstrating the ability of the gene to control genetic susceptibility to latent and active TB infection. Genetic variants of the *SP110* gene were investigated in the Taiwanese population (including 301 pulmonary TB patients, 68 LTBI individuals, and 278 healthy household contacts of the TB patients), and their association with susceptibility to latent and active TB infection was examined by performing an association analysis in a case-control study. We identified several SNPs (rs7580900, rs7580912, rs9061, rs11556887, and rs2241525) in the *SP110* gene that are associated with susceptibility to LTBI and/or TB disease. Our studies further showed that the same SNPs may have opposite effects on the control of susceptibility to LTBI versus TB. In addition, our analyses demonstrated that the *SP110* rs9061 SNP was associated with tumor necrosis factor-*α* (TNF*α*) levels in plasma in LTBI subjects. The results suggest that the polymorphisms within *SP110* have a role in controlling genetic susceptibility to latent and active TB infection in humans. To the best of our knowledge, this is the first report showing that the *SP110* variants are associated with susceptibility to LTBI. Our study also demonstrated that the identified *SP110* SNPs displayed the potential to predict the risk of LTBI and subsequent TB progression in Taiwan.

## 1. Introduction

Tuberculosis (TB), caused by *Mycobacterium tuberculosis* (*Mtb*) infection, remains one of the top ten causes of death in the world [1]. Approximately one-fourth of the world population has been infected with *Mtb* [2], but only 10% of these infected persons develop progressive disease during their lifetimes [3]. The majority of infected individuals remain healthy and noninfectious but carry *Mtb* in a latent form. Latent TB infection (LTBI) is a state during which a persistent host immune response to stimulation by *Mtb* antigens is sustained without evidence of clinically manifested active TB [4, 5]. As many as 10% of people with LTBI will go on to develop progressive disease in the near or remote future (a process named “TB reactivation”), and the risk is significantly higher in the presence of predisposing factors, such as coinfection with human immunodeficiency virus (HIV).

Persons with LTBI can be diagnosed by skin (tuberculin skin test (TST)) and/or blood (interferon-gamma (IFN*γ*) release assay (IGRA)) tests [6]. A positive result from these assays indicates an immune response to stimulation by *Mtb* antigens in the LTBI population, despite the fact that these individuals have a negative bacteriological test. However, neither TST nor IGRA can distinguish between LTBI and active TB disease or predict who will progress to active TB [7]. Given that the LTBI individuals represent a potential reservoir for future active TB cases, preventive therapy for LTBI is as important a goal as timely anti-TB treatment to reduce the burden of TB [8]. Therefore, identifying and treating cases with LTBI will contribute to TB elimination. Fundamental research for the development of diagnostic assays with improved performance and predictive assessment for TB reactivation will have practical applications and offer a substantial benefit for LTBI management.

In both humans and experimental animal models, genetic polymorphisms among hosts have been shown to contribute to the outcome of *Mtb* infection [9–13]. In mice, the *Ipr1* (intracellular pathogen resistance 1) gene is located within the *sst1* (supersusceptibility to tuberculosis 1) locus on chromosome 1 (49-54 cM) [14] and has been identified as a genetic determinant conferring host innate immunity to *Mtb* infection [15]. The previous studies indicate that the *Ipr1* gene may function to integrate mechanisms on controlling cell death, innate immunity, and pathogenesis during intracellular pathogen infections [15, 16]. The gene orthologous to the mouse *Ipr1* in humans is *SP110*, located on chromosome 2q37.1. Expression of both *Ipr1* and *SP110* genes is intensively regulated by IFNs, suggesting that the function of both genes is related to the IFN-mediated immune response [17].

Genetic defects in the *SP110* gene have been found to be responsible for hepatic veno-occlusive disease and immunodeficiency [18, 19], indicating that the gene plays important roles in immunity [20]. The *SP110* gene encodes the SP110 nuclear body protein, which has at least three isoforms, including the dominantly expressed SP110a, b, and c isoforms that are believed to be the result of alternative mRNA splicing. Our recent study demonstrated that SP110b, which is most similar to mouse Ipr1 and is expressed more abundantly than SP110a and SP110c, modulates nuclear factor-*κ*B (NF-*κ*B) activity resulting in the downregulation of tumor necrosis factor-*α* (TNF*α*) production and concomitant upregulation of NF-*κ*B-induced antiapoptotic gene expression, thereby suppressing IFN*γ*-mediated monocyte/macrophage cell death [21]. This indicates that the protein is crucial in the control of the activation of macrophages, the reservoir for *Mtb* persistence.

Although a number of genetic variants of the *SP110* gene have been reported to be associated with susceptibility to human TB, the results of studies regarding the relationship between *SP110* polymorphisms and TB susceptibility are inconsistent [22–29]. A family-based study in West Africa identified 3 *SP110* polymorphisms that are associated with TB susceptibility [22]; however, no significant associations between SP110 and disease susceptibility were identified by other, larger case-control studies conducted on various populations [24–26]. After screening Taiwanese populations for polymorphisms in *SP110*, we identified some single-nucleotide polymorphisms (SNPs) in *SP110* that are significantly associated with susceptibility to LTBI as well as TB disease. These results suggest that the *SP110* variants may provide novel predictive markers for TB infection status and disease outcome.

## 2. Materials and Methods

### 2.1. Human Subject Study

The study was conducted in accordance with the terms of the informed consent that was provided to, and received from, participants prior to inclusion in the study. This study was approved by the National Taiwan University Hospital Institutional Review Board (IRB No. 200612009M and IRB No. 201512169RINA). Human blood was ethically collected from patients with culture-confirmed pulmonary TB and their household contacts as described previously [30]. Briefly, the participating contacts received chest radiography, and mycobacteriology studies were conducted for 3 sputum samples (including acid-fast smear and mycobacterial culture) to exclude the possibility of active TB disease. Because routine BCG vaccination for newborns in Taiwan could affect the accuracy of tuberculin skin test, all enrolled contacts were then tested for LTBI using a T-SPOT.*TB* assay (Oxford Immunotec Ltd., Abingdon, UK) or QuantiFERON-TB Gold In-Tube assay (QFT) (Qiagen, Hilden, Germany), and the assays were interpreted according to the manufacturers' criteria. Both patients and contacts were excluded if they were tested positive for HIV infection. In total, 301 pulmonary TB patients, 68 individuals with LTBI, and 278 healthy household contacts were included in the study, and genomic DNA was extracted from their peripheral blood (1-2 mL) using a kit from Qiagen according to the manufacturer's protocol. *SP110* polymorphisms were identified from the National Center for Biotechnology Information dbSNP database (https://www.ncbi.nlm.nih.gov/snp). The SNPs were genotyped using the MassARRAY System (Sequenom, San Diego, CA, US), and the primer extension products were analyzed by MALDI-TOF mass spectrometry as previously described [31, 32]. Details of the primers that were used are listed in Table S1 in the Supplementary Materials.

Plasma samples were prepared from blood samples by centrifugation and then stored at −80°C until analysis. The TNF*α* levels in plasma samples were determined by a MAGPIX® platform (Luminex Corp., Austin, TX. US) with a MILLIPLEX MAP Human Cytokine/Chemokine Magnetic Bead Panel I kit (Merck Millipore, Billerica, MA, US) according to the manufacturer's instructions.

### 2.2. Statistical Analyses

The associations of gene polymorphisms with LTBI and TB disease were analyzed by SAS 9.4 software (SAS Institute, Cary, NC, US) [33]. Linkage disequilibrium and haplotype analyses were performed using Haploview (https://www.broadinstitute.org/haploview) [34]. Chi-square tests were used to compare frequencies. Odds ratios (ORs) were calculated with 95% confidence intervals (CIs). Bonferroni correction or false discovery rate (FDR) correction was applied for multiple comparison adjustments as indicated. The difference of the TNF*α* levels in plasma between two genotype groups of samples was calculated using a two-tailed unpaired *t*-test with Prism software (GraphPad, San Diego, CA, US). *p* values less than 0.05 were considered to indicate statistical significance, and the number of asterisks represents the degree of significance with regard to the *p* values.

## 3. Results

### 3.1. Association of Polymorphisms in *SP110* with LTBI and TB Susceptibility

To investigate the association between the *SP110* gene and control of *Mtb* infection, we examined polymorphisms in the gene of members of the Taiwanese population for genetic association with TB disease status. In this study, 301 pulmonary TB patients (202 males and 99 females; mean age: 63.1 ± 19.9 years), 68 individuals with LTBI (35 males and 33 females; mean age: 46.9 ± 17.7 years), and 278 healthy household contacts of the patients (92 males and 186 females; mean age: 47.1 ± 17.3 years) were included (Table 1). In total, 20 SNPs in the SP110 gene were selected for analysis. Of these, 10 SNPs that were not polymorphic or had a minor allele frequency less than 1% were not further included in the analysis, and the remaining 10 SNPs were then analyzed. We found that 3 SNPs (rs7580912, *p* = 0.0426, OR: 1.52, 95% CI: 1.01–2.39; rs7580900, *p* = 0.008, OR: 1.68, 95% CI: 1.14–2.48; and rs9061, *p* = 0.0026, OR: 0.39, 95% CI: 0.21–0.73) showed differential allele frequency distributions in LTBI cases vs. healthy controls (Table 2). After Bonferroni correction, rs9061 remained significant in this analysis (*p* < 0.05). Although allele frequencies of none of the 10 SNPs differed significantly in TB patients vs. healthy controls (Table 2), 3 SNPs (rs7580900, *p* = 0.0319, OR: 0.66, 95% CI: 0.45–0.97; rs9061, *p* = 0.0116, OR: 2.21, 95% CI: 1.18–4.15; and rs2241525, *p* = 0.0309, OR: 0.6, 95% CI: 0.38–0.96) exhibited differential allele frequency distributions in TB cases vs. LTBI individuals (Table 2). All of these SNPs were in accordance with the Hardy-Weinberg equilibrium (HWE).

The associations between *SP110* genotypes and susceptibility to LTBI and TB were then analyzed. In LTBI cases vs. healthy controls, we found that genotypes “GG” in rs7580912 (*p* = 0.025, OR: 2.451, 95% CI: 1.12–5.364) and “GG” in rs7580900 (*p* = 0.015, OR: 2.584, 95% CI: 1.208–5.53) were associated with LTBI risk and that genotype “GA” in rs9061 exhibited a protective effect on LTBI (*p* = 0.044, OR: 0.494, 95% CI: 0.239–0.981) (Table 3). We also found that genotypes “GG” in rs7580912 (*p* = 0.02, OR: 0.392, 95% CI: 0.179–0.86) and “GG” in rs7580900 (*p* = 0.017, OR: 0.392, 95% CI: 0.182–0.848) in TB patients vs. LTBI cases (Table 3), as well as “CT” in rs11556887 (*p* = 0.039, OR: 0.626, 95% CI: 0.401–0.976) in TB patients vs. healthy controls had a protective effect on TB (Table 3). These results indicated that several SNPs (rs7580900, rs7580912, rs9061, and rs11556887) in *SP110* were associated with susceptibility to LTBI and/or TB disease.

### 3.2. Association Analyses in Various Inheritance Models

The minor allele of each SNP was presumed as a risk factor compared to the major allele, and the associations between SNPs and susceptibility to LTBI and TB were analyzed in various inheritance models (Table 4). We found that in LTBI cases vs. healthy controls, rs9061 showed a protective effect on LTBI in both additive (*p* = 0.0059, OR: 0.41, 95% CI: 0.22–0.78) and dominant (*p* = 0.0112, OR: 0.42, 95% CI: 0.21–0.82) models, while rs7580900 was associated with LTBI risk in both additive (*p* = 0.0147, OR: 1.62, 95% CI: 1.1–2.39) and recessive (*p* = 0.0195, OR: 2.13, 95% CI: 1.13–4.03) models. In addition to this finding, rs7580912 was also associated with LTBI risk in a recessive model (*p* = 0.0142, OR: 2.49, 95% CI: 1.2–5.18). In TB patients vs. LTBI cases, rs9061 was associated with TB risk in both additive (*p* = 0.015, OR: 2.17, 95% CI: 1.16–4.06) and dominant (*p* = 0.0273, OR: 2.14, 95% CI: 1.09–4.22) models, while rs7580900 exhibited a protective effect on TB in both additive (*p* = 0.0261, OR: 0.64, 95% CI: 0.43–0.95) and recessive (*p* = 0.0117, OR: 0.45, 95% CI: 0.24–0.84) models and rs2241525 was associated with protection from TB in both additive (*p* = 0.0407, OR: 0.62, 95% CI: 0.4–0.98) and dominant (*p* = 0.0389, OR: 0.55, 95% CI: 0.31–0.97) models. Additionally, rs7580912 had a protective effect on TB in a recessive model (*p* = 0.0043, OR: 0.35, 95% CI: 0.17–0.72). After false discovery rate (FDR) correction, rs7580900 and rs7580912 remained significant (*p* = 0.041 and 0.0301, respectively) in a recessive model in this analysis.

### 3.3. Linkage Disequilibrium and Haplotype Analyses

We then examined linkage disequilibrium (LD) with the SNP markers in the *SP110* gene using a Haploview analysis. We found that the “ATATACGCGG” and “ATGTACGCGA” haplotypes met statistical significance (*p* = 0.0193, OR: 2.11, 95% CI: 1.11–3.99 and *p* = 0.0225, OR: 2.76, 95% CI: 1.12–6.84, respectively) for association with LTBI risk in LTBI cases vs. healthy controls (see Figure S1 in the Supplementary Materials) and that the “ATGTACGCGA” and “ATGAAAGCGA” haplotypes were statistically significant (*p* = 0.03, OR: 0.38, 95% CI: 0.16–0.95 and *p* = 0.0324, OR: 0.24, 95% CI: 0.06–0.99, respectively) with a protective effect on TB in TB cases vs. LTBI individuals (see Figure S2 in the Supplementary Materials). Interestingly, the “GCGTACGCGG” haplotype was associated with TB risk (*p* = 0.0169, OR: 3.81, 95% CI: 1.18–12.31) in TB cases vs. healthy controls (see Figure S3 in the Supplementary Materials), although none of the SNPs studied show a significant difference in frequency distributions in this comparison (Table 2). Noteworthily, when we compared “ATGTACGCAA” (the most frequent haplotype) with “ATGTACGCGA” (the haplotype that was associated with LTBI risk in LTBI cases vs. healthy controls and had a protective effect on TB in TB cases vs. LTBI individuals), the latter was found to be significantly affected by the rs7580900 SNP in both comparisons (*p* = 0.0225, OR: 2.76, 95% CI: 1.12–6.84 for LTBI cases vs. healthy controls; *p* = 0.03, OR: 0.38, 95% CI: 0.16–0.95 for TB cases vs. LTBI individuals) (see Figures S1 and S2 in the Supplementary Materials).

**Figure 1:**
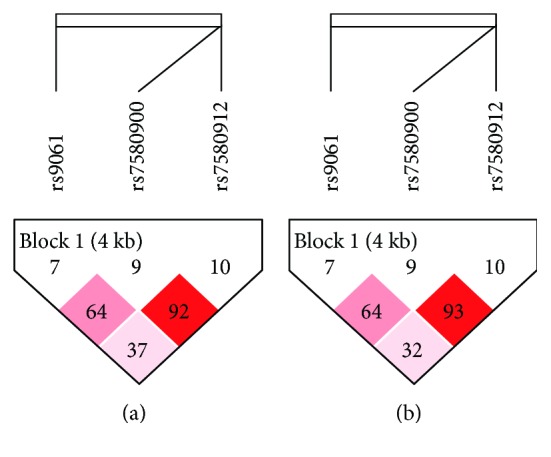
Haplotype block maps for *SP110* with 3 SNPs (rs9061, rs7580900, and rs7580912). The haplotype blocks were analyzed in LTBI cases vs. healthy controls (a) and TB cases vs. LTBI individuals (b), respectively.

We analyzed a block that includes 3 SNPs (rs9061, rs7580900, and rs7580912) in two comparisons (LTBI cases vs. healthy controls and TB cases vs. LTBI individuals) (Figure 1). We found that the “GAA” (*p* = 0.0037, OR: 1.77, 95% CI: 1.20–2.60), “GGG” (*p* = 0.0009, OR: 2.06, 95% CI: 1.34–3.17), “GGA” (*p* = 0.0009, OR: 2.81, 95% CI: 1.49–5.27), and “GAG” (*p* = 0.0001, OR: 10, 95% CI: 2.36–42.32) haplotypes were associated with disease risk and that the “AGG” haplotype had a protective effect on LTBI (*p* = 0.0254, OR: 0.41, 95% CI: 0.18–0.92) for LTBI cases vs. healthy controls (Table 5). In addition, the “GAA” (*p* = 0.0111, OR: 0.61, 95% CI: 0.42–0.90), “GGG” (*p* = 0.0408, OR: 0.65, 95% CI: 0.43–0.98), “GGA” (*p* = 0.0001, OR: 0.3, 95% CI: 0.16–0.57), and “GAG” (*p* = 0.0001, OR: 0.1, 95% CI: 0.02–0.4) haplotypes showed a protective effect on TB disease for TB patients vs. LTBI individuals (Table 5). These genetic studies suggest that the *SP110* gene plays a key role in modulating susceptibility to latent and active TB infection.

### 3.4. Association between the *SP110* rs9061 SNP and the TNF*α* Production in LTBI Subjects

**Figure 2:**
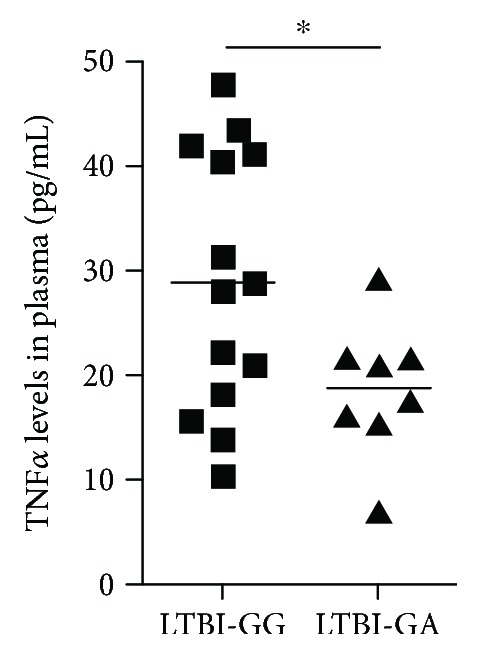
Association between the *SP110* rs9061 SNP and the TNF levels in plasma of LTBI subjects. The TNF production was determined in plasma samples from LTBI cases carrying the different genotypes of the rs9061 SNP by a MAGPIX instrument with a MILLIPLEX MAP Human Cytokine/Chemokine Magnetic Bead Panel I kit. Sample numbers are 14 for “GG” and 8 for “GA” genotypes, respectively. Statistical significance was calculated using a two-tailed unpaired *t*-test. ^∗^ *p* < 0.05.

TNF*α* plays a crucial role in controlling *Mtb* infection and TB reactivation; however, overproduction of TNF*α* may cause pathology [35, 36]. Our previous studies demonstrated that the SP110b protein, which is encoded by the *SP110* gene and whose expression is upregulated by IFNs, downregulates TNF*α* production in monocyte/macrophage cells activated by IFN*γ*, thereby alleviating cell death [21]. This finding indicates that the protein functions as a regulator of proinflammatory cytokines of host immunity contributing to a reduction in tissue damage caused by excessive inflammation [21]. To further investigate the potential association between the *SP110* SNPs and disease status, we next analyzed clinical parameters in the studied subjects. We measured TNF*α* levels in plasma from LTBI individuals who carry the different genotypes of the *SP110* SNPs and demonstrated that the “GA” genotype of rs9061 in LTBI individuals was associated with lower TNF*α* levels in plasma compared to “GG” LTBI subjects (Figure 2). The TNF*α* levels of healthy controls with both genotypes were undetectable (not shown). These data are in agreement with the protective role of the “GA” genotype of rs9061 in LTBI (Table 3) and further support our recent finding showing that the SP110b protein prevents cell death and tissue damage by downregulating TNF*α* production.

## 4. Discussion

*SP110* is strongly regulated by IFNs [17], suggesting its possible role in microbial immunity. Although many groups have studied the associations between the gene and TB susceptibility in a variety of populations, these studies show inconclusive results [22–29]. In our study, we recruited healthy household contacts of TB patients as controls, as these contacts are at a high risk of exposure to *Mtb*. It has been reported that approximately 80-100% of the contacts may have *Mtb* infection, and on average, 20% of them may develop disease [37], indicating that household contacts are at high risk of LTBI and active TB disease. Therefore, examination of this group carries a considerable importance for prevention and control of TB disease. To the best of our knowledge, this is the first study to demonstrate an association between *SP110* and LTBI, and it is the first study of the gene in the Taiwanese population. This work will help clarify the relationship between genetic variation in *SP110* with latent and active TB infection in an Asian population.

Several SNPs (rs7580900, rs7580912, rs9061, rs11556887, and rs2241525) in *SP110* showed an association with susceptibility to LTBI and/or TB disease in our study. In Table 3, we found that genotypes “GG” in rs7580912 (*p* = 0.025, OR: 2.451, 95% CI: 1.12–5.364) and “GG” in rs7580900 (*p* = 0.015, OR: 2.584, 95% CI: 1.208–5.53) were associated with LTBI susceptibility in LTBI cases vs. healthy controls, while the same SNP genotypes exhibited a protective effect on LTBI (“GG” in rs7580912, *p* = 0.02, OR: 0.392, 95% CI: 0.179–0.86; “GG” in rs7580900, *p* = 0.017, OR: 0.392, 95% CI: 0.182–0.848) in TB patients vs. LTBI individuals. In the Haploview analysis, we also found that the haplotype of multiple SNPs “ATGTACGCGA” in the *SP110* gene was significantly associated with LTBI risk (*p* = 0.0225, OR: 2.76, 95% CI: 1.12–6.84) in LTBI cases vs. healthy controls, while the same haplotypes had a protective effect on TB disease (*p* = 0.03, OR: 0.38, 95% CI: 0.16–0.95) in LTBI individuals vs. TB patients (see Figures S1 and S2 in the Supplementary Materials). In addition, 3 SNPs (rs9061, rs7580900, and rs7580912) with the haplotype “GAA” (*p* = 0.0037, OR: 1.77, 95% CI: 1.20–2.60), “GGG” (*p* = 0.0009, OR: 2.06, 95% CI: 1.34–3.17), “GGA” (*p* = 0.0009, OR: 2.81, 95% CI: 1.49–5.27), and “GAG” (*p* = 0.0001, OR: 10, 95% CI: 2.36–42.32) were associated with disease risk in LTBI cases vs. healthy controls, while the same haplotypes showed a protective effect on TB disease for TB patients vs. LTBI individuals (*p* = 0.0111, OR: 0.61, 95% CI: 0.42–0.90 for “GAA”; *p* = 0.0408, OR: 0.65, 95% CI: 0.43–0.98 for “GGG”; *p* = 0.0001, OR: 0.3, 95% CI: 0.16–0.57 for “GGA”; and *p* = 0.0001, OR: 0.1, 95% CI: 0.02–0.4 for “GAG”) (Table 5). These results revealed that the same SNP genotypes or haplotypes in the *SP110* gene had opposite effects on the control of susceptibility to LTBI and TB disease, suggesting that the gene may have differential roles in the control of susceptibility to LTBI and TB disease.

SNP rs9061 (G→A) introduces an amino acid change from glutamic acid to lysine at codon position 207 of the SP110 protein. Transforming an acidic amino acid to a basic amino acid may alter the protein structure or posttranslational modification of the SP110 protein leading to better downregulation of TNF*α* production. It has been suggested that the A allele may cause changes in *α*-helices and *β*-sheets in the secondary structure of the SP110 protein compared with the G allele [38]. In addition, we analyzed the SP110 protein using the ELM database (http://elm.eu.org/) and found that this amino acid change generates a potential binding motif for the C-terminal ubiquitin-like domain (CTD) of ubiquitin specific protease 7 (USP7), one of the most abundant deubiquitinases [39]. USP7 plays important roles in various biological activities, including cell survival, proliferation, apoptosis, and tumorigenesis [40, 41]. As shown in Figure 2, the data demonstrated an association between the “GA” genotype of rs9061 with lower TNF*α* levels in plasma from LTBI individuals compared to “GG” LTBI subjects. One possible explanation for this result is that SP110 with the “GA” genotype at rs9061 may interact with USP7 and thus is more stable than the SP110 with the “GG” genotype, resulting in a more efficient downregulation of TNF*α* production. However, further functional studies are needed to elucidate the exact effects of SNP rs9061 on the *SP110* gene and disease risk.

Individuals with LTBI, based on a positive result in the TST or the IGRA, usually show no disease symptoms and acquire an effective adaptive immunity. However, a proportion of people with LTBI might reactivate and develop clinical disease. The positive results from these assays indicate an immune response to stimulation by *Mtb* antigens in the LTBI population; however, neither TST nor IGRA can distinguish between LTBI and active TB. In addition, these assays also cannot predict which LTBI cases will progress to active TB [7]. To control and eliminate TB, worldwide TB eradication endeavors have been focused on identifying and treating cases with LTBI. Therefore, our studies identified several SNPs in *SP110* that are significantly associated with controlling susceptibility to LTBI and TB disease and thus may provide novel predictive markers for latent and active TB infection. This study may also yield an improved strategy that can identify persons at increased risk of the disease.

There were some limitations in our study. First, this study dealt with relatively small sample sizes (301, 68, and 278 for TB patients, LTBI cases, and healthy household contacts, respectively). Further large-scale studies are, therefore, needed to validate the predictive value of the *SP110* SNPs identified in this study. Second, the LTBI number of our study group is small, and besides, the results on plasma TNF*α* levels were analyzed from groups with even smaller sample sizes; therefore, the difference in TNF*α* levels in plasma of LTBI cases with two genotypes may be underestimated and should be verified in a different population. In addition, the analysis of TNF*α* levels in plasma of active TB patients with the different genotypes is in progress. The result may help support our finding in LTBI individuals and clarify the potential association between the *SP110* SNPs and disease status.

## 5. Conclusions

The results suggest that the *SP110* variants have a role in controlling genetic susceptibility to latent and active TB infection in humans. To the best of our knowledge, this is the first report demonstrating associations of polymorphisms in *SP110* with LTBI susceptibility. Additionally, we provide evidence that the *SP110* rs9061 SNP is associated with plasma TNF*α* levels in LTBI individuals. These data suggest that the identified *SP110* SNPs may serve as a biomarker for latent and active TB infection in Taiwan.

## Figures and Tables

**Table 1:** Demographic characteristics of TB patients, LTBI cases, and healthy controls in this study.

Group	Number			Age (years)	
	Total	Male (%)	Female (%)	Mean ± SD	Range
Health	278	92 (33)	186 (67)	47.1 ± 17.3	15.2-93.9
LTBI	68	35 (51)	33 (49)	46.9 ± 17.7	19.5-86.5
TB	301	202 (67)	99 (33)	63.1 ± 19.9	19.4-98.7

**Table:** (a) Allele frequencies in LTBI cases and healthy controls and odds ratio estimates for LTBI

SNP ID	Position^1^	Location^2^	HWEp	Alleles	LTBI *n* (%)	Health *n* (%)	OR (95% CI)	*p* value
rs7580912	230216690	Intron 2-3	0.1113	A	48 (40)	165 (30.4)	1.52 (1.01-2.39)	*0.0426*
				G	72 (60)	377 (69.6)		
rs7580900	230216669	Intron 2-3	0.5446	A	67 (52.3)	214 (39.5)	1.68 (1.14-2.48)	*0.008*
				G	61 (47.7)	328 (60.5)		
rs11556887	230212961	Exon 4	0.0593	C	113 (88.3)	476 (87.8)	0.96 (0.53-1.74)	0.8862
				T	15 (11.7)	66 (12.2)		
rs9061	230212395	Exon 5	0.1272	G	118 (90.8)	427 (79.4)	0.39 (0.21-0.73)	*0.0026* ^∗^
				A	12 (9.2)	111 (20.6)		
rs3820974	230211574	Intron 5-6	0.8257	C	93 (71.5)	366 (67.5)	0.83 (0.54-1.26)	0.3774
				A	37 (28.5)	176 (32.5)		
rs1365776	230207994	Exon 8	0.4341	A	111 (91)	479 (89.7)	0.86 (0.44-1.70)	0.6708
				G	11 (9)	55 (10.3)		
rs41309108	230201006	Intron 9-10	0.0333	T	105 (80.8)	415 (76.6)	0.78 (0.48-1.26)	0.3039
				A	25 (19.2)	127 (23.4)		
rs2241525	230178086	Intron 13-14	0.451	G	30 (24.6)	93 (17.4)	1.55 (0.97-2.47)	0.067
				A	92 (75.4)	441 (82.6)		
rs1135791	230177560	Exon 14	0.4826	T	107 (89.2)	472 (87.4)	0.76 (0.41-1.43)	0.5952
				C	13 (10.8)	68 (12.6)		
rs10498244	230173117	Intron 14-15	0.5064	A	95 (89.6)	459 (86.6)	0.75 (0.38-1.47)	0.3972
				G	11 (10.4)	71 (13.4)		

^1^NCBI Reference Sequence: NC_000002.12. ^2^Based on SP110c (NCBI Reference Sequence: NM_080424.2). HWE: Hardy-Weinberg equilibrium; OR: odds ratio; CI: confidence interval; ORs are adjusted for gender. The significant ORs are shown in italic. ^∗^OR remains significant after Bonferroni correction.

**Table:** (b) Allele frequencies in TB patients and healthy controls and odds ratio estimates for TB

SNP ID	Position^1^	Location^2^	HWEp	Alleles	LTBI *n* (%)	Health *n* (%)	OR (95% CI)	*p* value
rs7580912	230216690	Intron 2-3	0.9813	A	201 (33.6)	165 (30.4)	1.16 (0.91-1.49)	0.2524
				G	397 (66.4)	377 (69.6)		
rs7580900	230216669	Intron 2-3	0.8338	A	251 (42)	214 (39.5)	1.11 (0.88-1.41)	0.393
				G	347 (58)	328 (60.5)		
rs11556887	230212961	Exon 4	0.7296	C	543 (90.8)	476 (87.8)	0.73 (0.50-1.07)	0.1028
				T	55 (9.2)	66 (12.2)		
rs9061	230212395	Exon 5	0.0162	G	485 (81.6)	427 (79.4)	0.86 (0.64-1.16)	0.3326
				A	109 (18.4)	111 (20.6)		
rs3820974	230211574	Intron 5-6	0.9602	C	195 (32.6)	17 (32.5)	1.01 (0.79-1.29)	0.9609
				A	403 (67.4)	366 (67.5)		
rs1365776	230207994	Exon 8	0.0396	A	67 (11.2)	55 (10.3)	1.10 (0.75-1.59)	0.6242
				G	531 (88.8)	479 (89.7)		
rs41309108	230201006	Intron 9-10	0.0393	T	463 (77.4)	415 (76.6)	0.95 (0.72-1.26)	0.7314
				A	135 (22.6)	127 (23.4)		
rs2241525	230178086	Intron 13-14	0.102	G	495 (83.6)	441 (82.6)	0.93 (0.68-1.27)	0.6447
				A	97 (16.4)	93 (17.4)		
rs1135791	230177560	Exon 14	0.3517	T	92 (15.4)	68 (12.6)	1.28 (0.92-1.80)	0.176
				C	506 (84.6)	472 (87.4)		
rs10498244	230173117	Intron 14-15	0.5808	A	88 (14.7)	71 (13.4)	1.13 (0.81-1.59)	0.525
				G	510 (85.3)	459 (86.6)		

^1^NCBI Reference Sequence: NC_000002.12. ^2^Based on SP110c (NCBI Reference Sequence: NM_080424.2). HWE: Hardy-Weinberg equilibrium; OR: odds ratio; CI: confidence interval; ORs are adjusted for gender.

**Table:** (c) Allele frequencies in TB patients and LTBI cases and odds ratio estimates for TB

SNP ID	Position^1^	Location^2^	HWEp	Alleles	LTBI *n* (%)	Health *n* (%)	OR (95% CI)	*p* value
rs7580912	230216690	Intron 2-3	0.7351	A	397 (66.4)	72 (60)	0.76 (0.51-1.14)	0.1797
				G	201 (33.6)	48 (40)		
rs7580900	230216669	Intron 2-3	0.9968	A	347 (58)	61 (47.7)	0.66 (0.45-0.97)	*0.0319*
				G	251 (42)	67 (52.3)		
rs11556887	230212961	Exon 4	0.8656	C	543 (90.8)	113 (88.3)	0.76 (0.42-1.40)	0.3804
				T	55 (9.2)	15 (11.7)		
rs9061	230212395	Exon 5	0.0995	G	109 (18.4)	12 (9.2)	2.21 (1.18-4.15)	*0.0116*
				A	485 (81.6)	118 (90.8)		
rs3820974	230211574	Intron 5-6	0.8727	C	195 (32.6)	37 (28.5)	1.22 (0.80-1.85)	0.3577
				A	403 (67.4)	93 (71.5)		
rs1365776	230207994	Exon 8	0.028	A	67 (11.2)	11 (9)	1.27 (0.65-2.49)	0.4786
				G	531 (88.8)	111 (91)		
rs41309108	230201006	Intron 9-10	0.2174	T	135 (22.6)	25 (19.2)	1.22 (0.76-1.97)	0.4039
				A	463 (77.4)	105 (80.8)		
rs2241525	230178086	Intron 13-14	0.1275	G	495 (83.6)	92 (75.4)	0.60 (0.38-0.96)	*0.0309*
				A	97 (16.4)	30 (24.6)		
rs1135791	230177560	Exon 14	0.3733	T	92 (15.4)	13 (10.8)	1.50 (0.81-2.77)	0.1979
				C	506 (84.6)	107 (89.2)		
rs10498244	230173117	Intron 14-15	0.8897	A	88 (14.7)	11 (10.4)	1.49 (0.77-2.89)	0.2363
				G	510 (85.3)	95 (89.6)		

^1^NCBI Reference Sequence: NC_000002.12. ^2^Based on SP110c (NCBI Reference Sequence: NM_080424.2). HWE: Hardy-Weinberg equilibrium; OR: odds ratio; CI: confidence interval; ORs are adjusted for gender. The significant ORs are shown in italic.

**Table:** (a) Association between *SP110* SNP genotypes and LTBI risk in LTBI cases vs. healthy controls

SNP ID	Genotypes	Health no. (%)	LTBI no. (%)	OR (95% CI)	*p* value
rs7580912	AA	134 (49)	26 (43)	Ref	—
	GA	111 (41)	20 (33)	0.884 (0.464-1.686)	0.709
	GG	27 (10)	14 (23)	2.451 (1.12-5.364)	*0.025*
rs7580900	AA	100 (37)	16 (25)	Ref	—
	GA	128 (47)	29 (45)	1.403 (0.716-2.749)	0.324
	GG	43 (16)	19 (30)	2.584 (1.208-5.53)	*0.015*
rs11556887	CC	206 (76)	49 (77)	Ref	—
	CT	64 (24)	15 (23)	0.982 (0.513-1.882)	0.957
	TT	1 (0.4)	0 (0)	<0.001 (<0.001-999.999)	0.967
rs9061	GG	177 (65)	54 (82)	Ref	—
	AA	16 (6)	0 (0)	<0.001 (<0.001-999.999)	0.901
	GA	79 (29)	12 (18)	0.494 (0.239-0.981)	***0.044***
rs3820974	CC	125 (46)	34 (52)	Ref	—
	AA	29 (11)	6 (9)	0.712 (0.269-1.886)	0.494
	CA	118 (43)	25 (38)	0.703 (0.391-1.264)	0.239
rs1365776	AA	217 (81)	52 (83)	Ref	—
	GA	47 (18)	10 (16)	0.757 (0.345-1.66)	0.487
	GG	4 (1)	1 (1)	1.212 (0.129-11.39)	0.867
rs41309108	TT	153 (56)	41 (63)	Ref	—
	AA	10 (4)	1 (1)	0.395 (0.048-3.248)	0.387
	TA	109 (40)	23 (35)	0.768 (0.433-1.362)	0.367
rs2241525	GG	186 (69)	36 (58)	Ref	—
	AA	10 (4)	4 (7)	1.984 (0.579-6.802)	0.276
	GA	75 (28)	22 (35)	1.509 (0.828-2.75)	0.179
rs1135791	TT	206 (75)	48 (79)	Ref	—
	CC	3 (1)	0 (0)	<0.001 (<0.001-999.999)	0.943
	CT	64	13 (21)	0.86 (0.434-1.703)	0.667
rs10498244	AA	199 (75)	44 (83)	Ref	—
	AG	61 (23)	7 (13)	0.523 (0.223-1.223)	0.135
	GG	5 (2)	2 (4)	1.9 (0.354-10.203)	0.454

OR: odds ratio; CI: confidence interval; ORs are adjusted for gender. The significant ORs are shown in italic.

**Table:** (b) Association between *SP110* SNP genotypes and TB risk in TB patients vs. LTBI cases

SNP ID	Genotypes	Health no. (%)	LTBI No. (%)	OR (95% CI)	*p* value
rs7580912	AA	26 (43)	129 (43)	Ref	—
	GA	20 (33)	139 (47)	1.377 (0.731-2.595)	0.322
	GG	14 (23)	31 (10)	0.392 (0.179-0.86)	*0.02*
rs7580900	AA	16 (25)	98 (33)	Ref	—
	GA	29 (45)	151 (50)	0.863 (0.444-1.878)	0.664
	GG	19 (30)	50 (17)	0.392 (0.182-0.848)	*0.017*
rs11556887	CC	49 (77)	248 (83)	Ref	—
	CT	15 (23)	47 (16)	0.628 (0.324-1.219)	0.169
	TT	0 (0)	4 (1)	>999.999 (<0.001->999.999)	0.988
rs9061	GG	54 (82)	203 (68)	Ref	—
	AA	0 (0)	15 (5)	>999.999 (<0.001->999.999)	0.974
	GA	12 (18)	79 (27)	1.831 (0.924-3.627)	0.083
rs3820974	CC	34 (52)	136 (45)	Ref	—
	AA	6 (9)	32 (11)	1.261 (0.483-3.293)	0.637
	CA	25 (38)	131 (44)	1.31 (0.739-2.323)	0.356
rs1365776	AA	52 (83)	240 (80)	Ref	—
	GA	10 (16)	51 (17)	1.264 (0.58-2.753)	0.556
	GG	1 (1)	8 (3)	2.028 (0.244-16.888)	0.513
rs41309108	TT	41 (63)	176 (59)	Ref	—
	AA	1 (1)	12 (4)	2.401 (0.3-19.219)	0.409
	TA	23 (35)	111 (37)	1.096 (0.621-1.932)	0.753
rs2241525	GG	36 (58)	211 (71)	Ref	—
	AA	4 (7)	12 (4)	0.47 (0.142-1.562)	0.218
	GA	22 (35)	73 (25)	0.564 (0.31-1.026)	0.061
rs1135791	TT	48 (79)	212 (71)	Ref	—
	CC	0 (0)	5 (2)	>999.999 (<0.001->999.999)	0.986
	CT	13 (21)	82 (27)	1.33 (0.68-2.602)	0.404
rs10498244	AA	44 (83)	215 (72)	Ref	—
	AG	7 (13)	80 (27)	2.137 (0.917-4.981)	0.079
	GG	2 (4)	4 (1)	0.441 (0.074-2.62)	0.368

OR: odds ratio; CI: confidence interval; ORs are adjusted for gender. The significant ORs are shown in italic.

**Table:** (c) Association between *SP110* SNP genotypes and TB risk in TB patients vs. healthy controls

SNP ID	Genotypes	Health no. (%)	LTBI no. (%)	OR (95% CI)	*p* value
rs7580912	AA	134 (49)	129 (43)	Ref	—
	GA	111 (41)	139 (47)	1.258 (0.869-1.822)	0.224
	GG	27 (10)	31 (10)	0.877 (0.474-1.622)	0.676
rs7580900	AA	100 (37)	98 (33)	Ref	—
	GA	128 (47)	151 (50)	1.258 (0.851-1.86)	0.249
	GG	43 (16)	50 (17)	1.023 (0.601-1.74)	0.934
rs11556887	CC	206 (76)	248 (83)	Ref	—
	CT	64 (23.6)	47 (16)	0.626 (0.401-0.976)	*0.039*
	TT	1 (0.4)	4 (1)	2.387 (0.243-23.458)	0.456
rs9061	GG	176 (65)	203 (68)	Ref	—
	AA	17 (6)	15 (5)	0.792 (0.36-1.742)	0.561
	GA	79 (29)	79 (27)	0.886 (0.597-1.313)	0.546
rs3820974	CC	125 (46)	136 (45)	Ref	—
	AA	29 (11)	32 (11)	0.871 (0.473-1.603)	0.657
	CA	118 (43)	131 (44)	0.942 (0.651-1.363)	0.749
rs1365776	AA	217 (81)	240 (80)	Ref	—
	GA	47 (18)	51 (17)	0.931 (0.582-1.489)	0.767
	GG	4 (2)	8 (3)	2.291 (0.631-8.322)	0.208
rs41309108	TT	153 (56)	176 (59)	Ref	—
	AA	9 (3)	12 (4)	0.982 (0.382-2.527)	0.971
	TA	109 (40)	111 (37)	0.852 (0.593-1.225)	0.387
rs2241525	GG	186 (69)	211 (71)	Ref	—
	AA	10 (3)	12 (4)	1.004 (0.402-2.505)	0.994
	GA	75 (28)	73 (25)	0.85 (0.569-1.268)	0.425
rs1135791	TT	206 (75)	212 (71)	Ref	—
	CC	3 (1)	5 (2)	1.681 (0.371-7.617)	0.5
	CT	64 (24)	82 (27)	1.177 (0.788-1.76)	0.426
rs10498244	AA	199 (75)	215 (72)	Ref	—
	AG	61 (23)	80 (27)	1.185 (0.787-1.785)	0.417
	GG	5 (2)	4 (1)	0.774 (0.191-3.133)	0.719

OR: odds ratio; CI: confidence interval; ORs are adjusted for gender. The significant ORs are shown in italic.

**Table:** (a) Association analyses of *SP110* SNP genotypes in an additive model

SNP ID	TB vs. health		TB vs. LTBI		LTBI vs. health	
	OR (95% CI)	*p* value	OR (95% CI)	*p* value	OR (95% CI)	*p* value
rs9061	0.89 (0.66,1.2)	0.4382	2.17 (1.16,4.06)	*0.015*	0.41 (0.22,0.78)	*0.0059*
rs7580900	1.07 (0.83,1.38)	0.5839	0.64 (0.43,0.95)	*0.0261*	1.62 (1.1,2.39)	*0.0147*
rs3820974	0.94 (0.72,1.23)	0.6661	1.2 (0.79,1.82)	0.4009	0.79 (0.51,1.21)	0.2761
rs41309108	0.9 (0.66,1.22)	0.4917	1.2 (0.73,1.97)	0.4824	0.73 (0.44,1.23)	0.2366
rs7580912	1.05 (0.8,1.37)	0.7307	0.74 (0.49,1.1)	0.1387	1.4 (0.94,2.08)	0.0992
rs1135791	1.2 (0.83,1.73)	0.3242	1.46 (0.77,2.77)	0.2444	0.81 (0.42,1.56)	0.525
rs1365776	1.1 (0.75,1.62)	0.6153	1.32 (0.7,2.5)	0.3933	0.84 (0.43,1.65)	0.6133
rs10498244	1.1 (0.77,1.59)	0.5919	1.4 (0.71,2.73)	0.3303	0.77 (0.4,1.5)	0.4457
rs2241525	0.91 (0.66,1.25)	0.5681	0.62 (0.4,0.98)	*0.0407*	1.46 (0.92,2.32)	0.1102
rs11556887	0.72 (0.48,1.08)	0.1155	0.75 (0.41,1.39)	0.362	0.96 (0.5,1.81)	0.8916

OR: odds ratio; CI: confidence interval; ORs are adjusted for gender. The significant ORs are shown in italic.

**Table:** (b) Association analyses of *SP110* SNP genotypes in a dominant model

SNP ID	TB vs. health		TB vs. LTBI		LTBI vs. health	
	OR (95% CI)	*p* value	OR (95% CI)	*p* value	OR (95% CI)	*p* value
rs9061	0.87 (0.6,1.26)	0.4646	2.14 (1.09,4.22)	*0.0273*	0.42 (0.21,0.82)	*0.0112*
rs7580900	1.22 (0.84,1.76)	0.2929	0.68 (0.37,1.27)	0.2276	1.74 (0.93,3.24)	0.083
rs3820974	0.93 (0.65,1.32)	0.6879	1.3 (0.76,2.24)	0.3378	0.71 (0.41,1.23)	0.2193
rs41309108	0.86 (0.6,1.23)	0.4092	1.15 (0.66,2.01)	0.6191	0.74 (0.42,1.3)	0.2917
rs7580912	1.18 (0.83,1.68)	0.3571	0.98 (0.55,1.72)	0.9312	1.2 (0.68,2.12)	0.5388
rs1135791	1.2 (0.81,1.78)	0.3678	1.41 (0.72,2.75)	0.3146	0.83 (0.42,1.64)	0.5948
rs1365776	1.03 (0.66,1.6)	0.9033	1.34 (0.64,2.82)	0.4408	0.79 (0.37,1.67)	0.5353
rs10498244	1.15 (0.77,1.72)	0.4807	1.75 (0.81,3.78)	0.1536	0.63 (0.29,1.35)	0.2341
rs2241525	0.87 (0.59,1.27)	0.4667	0.55 (0.31,0.97)	***0.0389***	1.57 (0.88,2.78)	0.1239
rs11556887	0.66 (0.43,1.02)	0.0609	0.67 (0.35,1.3)	0.2363	0.97 (0.51,1.86)	0.9282

OR: odds ratio; CI: confidence interval; ORs are adjusted for gender. The significant ORs are shown in italic.

**Table:** (c) Association analyses of *SP110* SNP genotypes in a recessive model

SNP ID	TB vs. health		TB vs. LTBI		LTBI vs. health	
	OR (95% CI)	*p* value	OR (95% CI)	*p* value	OR (95% CI)	*p* value
rs9061	0.82 (0.38,1.78)	0.6227	—	—	—	—
rs7580900	0.92 (0.57,1.48)	0.737	0.45 (0.24,0.84)	*0.0117*	2.13 (1.13,4.03)	*0.0195* ^∗^
rs3820974	0.92 (0.52,1.62)	0.7796	1.13 (0.45,2.83)	0.8018	0.84 (0.33,2.15)	0.7209
rs41309108	1.05 (0.41,2.66)	0.9241	2.35 (0.3,18.52)	0.4186	0.43 (0.05,3.55)	0.437
rs7580912	0.79 (0.44,1.42)	0.4367	0.35 (0.17,0.72)	*0.0043*	2.49 (1.2,5.18)	*0.0142* ^∗^
rs1135791	1.63 (0.36,7.51)	0.5284	—	—	—	—
rs1365776	2.25 (0.63,8.05)	0.2129	1.94 (0.23,16.11)	0.5375	1.24 (0.13,11.51)	0.8504
rs10498244	0.74 (0.18,3.05)	0.6804	0.38 (0.06,2.23)	0.2844	2.27 (0.42,12.19)	0.3385
rs2241525	1.05 (0.42,2.6)	0.9172	0.56 (0.17,1.83)	0.3374	1.73 (0.52,5.81)	0.3747
rs11556887	2.63 (0.27,25.7)	0.4064	—	—	—	—

OR: odds ratio; CI: confidence interval; ORs are adjusted for gender. The significant ORs are shown in italic. ^∗^ORs remain significant after false discovery rate (FDR) correction.

**Table:** (a) Association of haplotype frequencies with LTBI risk in LTBI cases and healthy controls

Haplotypes	Frequencies		Chi-square	OR (95% CI)	*p* value
	LTBI	Health			
GAA	0.417	0.558	8.417	1.77 (1.20-2.60)	*0.0037*
GGG	0.310	0.179	11.048	2.06 (1.34-3.17)	*9.00E − 04*
AGG	0.053	0.120	4.997	0.41 (0.18-0.92)	*0.0254*
GGA	0.134	0.052	10.99	2.81 (1.49-5.27)	*9.00E − 04*
AGA	0.021	0.043	1.429	0.47 (0.13-1.68)	0.232
AAA	0.019	0.042	1.579	0.44 (0.12-1.65)	0.209
GAG	0.047	0.005	14.5	10.00 (2.36-42.32)	*1.00E − 04*

OR: odds ratio; CI: confidence interval; ORs are adjusted for gender. The significant ORs are shown in italic.

**Table:** (b) Association of haplotype frequencies with TB risk in TB cases vs. LTBI individuals

Haplotypes	Frequencies		Chi-square	OR (95% CI)	*p* value
	TB	LTBI			
GAA	0.540	0.417	6.445	0.61 (0.42-0.90)	*0.0111*
GGG	0.228	0.313	4.184	0.65 (0.43-0.98)	*0.0408*
AGG	0.103	0.053	3.191	2.05 (0.91-4.62)	0.074
GGA	0.043	0.130	14.591	0.30 (0.16-0.57)	*0.0001*
AGA	0.045	0.021	1.564	2.16 (0.62-7.49)	0.211
AAA	0.035	0.018	0.993	1.95 (0.51-7.53)	0.3191
GAG	0.005	0.047	16.031	0.10 (0.02-0.40)	*0.0001*

OR: odds ratio; CI: confidence interval; ORs are adjusted for gender. The significant ORs are shown in italic.

## Data Availability

The data used to support the findings of this study are available from the corresponding author upon request.

## Supplementary Materials

Supplementary Materials Table S1: primers for genotype analysis for SP110. Figure S1: association of SP110 SNP haplotypes with LTBI risk in LTBI cases vs. healthy controls. (a) Haplotype block map for SP110 with 10 SNPs. (b) Association of haplotype frequencies with LTBI risk in LTBI cases and healthy controls. Figure S2: association of SP110 SNP haplotypes with TB risk in TB cases vs. LTBI individuals. (a) Haplotype block map for SP110 with 10 SNPs. (b) Association of haplotype frequencies with TB risk in TB cases vs. LTBI individuals. Figure S3: association of SP110 SNP haplotypes with TB risk in TB cases vs. healthy controls. (a) Haplotype block map for SP110 with 10 SNPs. (b) Association of haplotype frequencies with TB risk in TB cases and healthy controls.
