# Supplementary material for: SP110 Polymorphisms Are Genetic Markers for Vulnerability to Latent and Active Tuberculosis Infection in Taiwan
Source: Dis Markers. 2018 Dec 5;2018:4687380. doi: 10.1155/2018/4687380 (PMC6304864; doi:10.1155/2018/4687380)
Supplement: Supplementary Materials — Table S1: primers for genotype analysis for SP110. Figure S1: association of SP110 SNP haplotypes with LTBI risk in LTBI cases vs. healthy controls. (a) Haplotype block map for SP110 with 10 SNPs. (b) Association of haplotype frequencies with LTBI risk in LTBI cases and healthy controls. Figure S2: association of SP110 SNP haplotypes with TB risk in TB cases vs. LTBI individuals. (a) Haplotype block map for SP110 with 10 SNPs. (b) Association of haplotype frequencies with TB risk in TB cases vs. LTBI individuals. Figure S3: association of SP110 SNP haplotypes with TB risk in TB cases vs. healthy controls. (a) Haplotype block map for SP110 with 10 SNPs. (b) Association of haplotype frequencies with TB risk in TB cases and healthy controls. [file 4687380.f1.pdf]

a

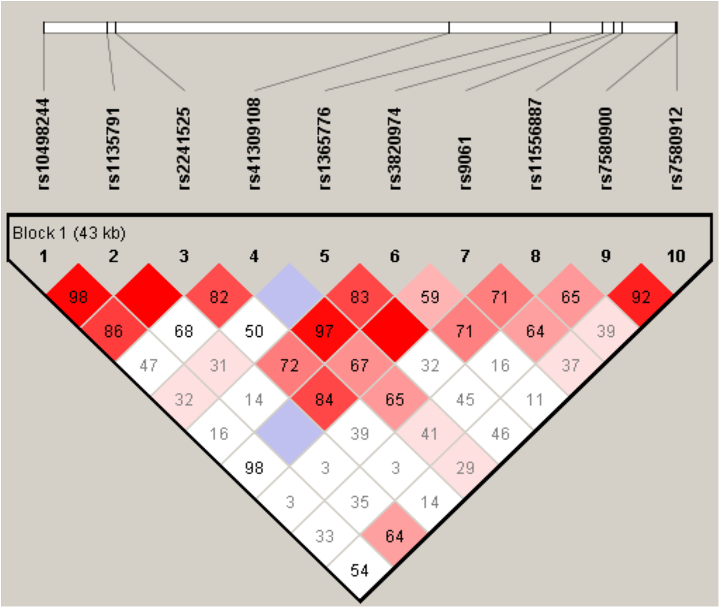

b

Haplotypes	Frequencies		Chi Square	OR (95%CI)	*P* value
	LTBI	Health			
ATGTACGCAA	0.231,	0.281	1.3	0.77 (0.49-1.21)	0.2542
ATATACGCAA	0.049,	0.090	2.256	0.52 (0.22-1.23)	0.1331
ATGTACGCGG	0.099	0.068	1.441	1.50 (0.77-2.94)	0.23
ATATACGCGG	0.122	0.061	5.472	2.11 (1.11-3.99)	***0.0193***
GCGTACGCAA	0.028	0.060	1.981	0.46 (0.15-1.39)	0.1593
ATGAAAATGG	0.015	0.051	3.191	0.28 (0.06-1.25)	0.074
ATGAAAGCAA	0.025	0.048	1.327	0.50 (0.15-1.64)	0.2493
ATGAAAACGG	0.024	0.045	1.141	0.53 (0.16-1.76)	0.2854
ATGTGAGCAA	0.008	0.042	3.488	0.18 (0.02-1.36)	0.0618
ATGTACGCGA	0.063	0.024	5.206	2.76 (1.12-6.84)	***0.0225***
ATGAAAGCGG	0.041	0.021	1.73	2.00 (0.69-5.74)	0.1884
GCGTGAGCAA	0.024	0.025	0.008	0.95 (0.27-3.37)	0.9272
ATGTACACGA	0.007	0.020	0.933	0.35 (0.04-3.06)	0.334
ATGAAAATAA	0.010	0.019	0.577	0.48 (0.07-3.24)	0.4477
ATGAAAATGA	0.014	0.017	0.051	0.84 (0.17-4.19)	0.8216
GCGTACGCGA	0.018	0.010	0.563	1.79 (0.38-8.44)	0.4532
ATGTACACGG	0.008	0.011	0.092	0.69 (0.08-5.8)	0.7617

1 OR: odds ratio; CI: confidence interval; ORs are adjusted for gender. The significant ORs are shown in italic.

Figure S1: Association of *SP110* SNP haplotypes with LTBI risk in LTBI cases vs. healthy controls. **a** Haplotype block map for *SP110* with 10 SNPs. **b** Association of haplotype frequencies with LTBI risk in LTBI cases and healthy controls.

a

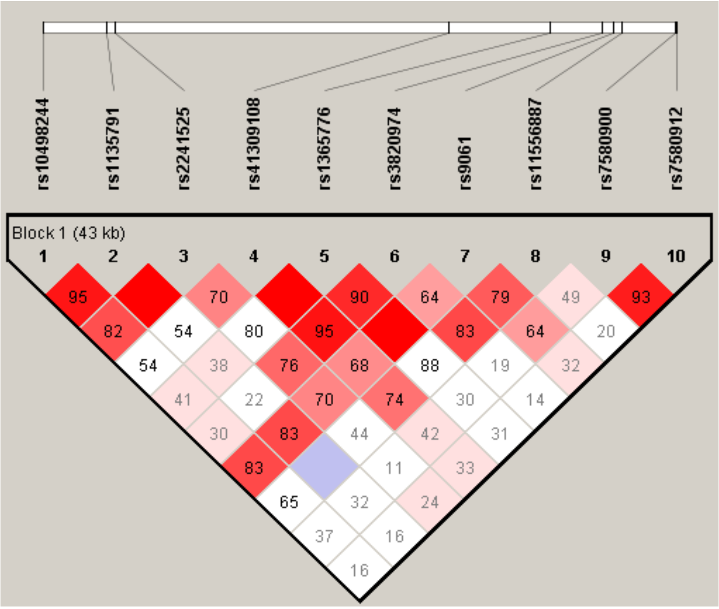

b

Haplotypes	Frequencies		Chi Square	OR (95%CI)	*P* value
	TB	LTBI			
ATGTACGCAA	0.304,	0.235	2.427	1.42 (0.91-2.22)	0.1193
ATATACGCAA	0.076,	0.051	1.03	1.56 (0.66-3.64)	0.3103
ATGTACGCGG	0.062,	0.092	1.554	0.65 (0.33-1.28)	0.2125
ATATACGCGG	0.058,	0.093	2.178	0.60 (0.30-1.19)	0.14
ATGAAAACGG	0.055,	0.024	2.11	2.35 (0.72-7.64)	0.1463
GCGTGAGCAA	0.046,	0.024	1.27	1.98 (0.59-6.63)	0.2598
GCGTACGCGG	0.040,	0.034	0.123	1.20 (0.42-3.41)	0.7257
ATGAAAGCGG	0.030,	0.050	1.351	0.58 (0.23-1.46)	0.2451
ATGTACGCGA	0.024,	0.060	4.708	0.38 (0.16-0.95)	***0.03***
ATGAAAGCAA	0.030,	0.023	0.204	1.33 (0.38-4.66)	0.6512
GCGTACGCAA	0.029,	0.016	0.67	1.81 (0.43-7.68)	0.413
ATGTGAGCAA	0.028,	0.008	1.844	3.71 (0.49-28.11)	0.1745
ATGTACACGA	0.024,	0.008	1.403	3.17 (0.41-24.25)	0.2362
ATGAAAATAA	0.023,	0.009	0.957	2.47 (0.38-16.13)	0.328
ATGAAAATGG	0.022,	0.014	0.312	1.55 (0.32-7.47)	0.5766
ATGTGAGCGG	0.020,	0.015	0.157	1.38 (0.30-6.43)	0.6921
ATGAAAATGA	0.020,	0.014	0.21	1.44 (0.30-6.99)	0.6468
ATGAAAGCGA	0.007,	0.028	4.576	0.24 (0.06-0.99)	***0.0324***

1 OR: odds ratio; CI: confidence interval; ORs are adjusted for gender. The significant ORs are shown in italic.

Figure S2: Association of *SP110* SNP haplotypes with TB risk in TB cases vs. LTBI individuals. **a** Haplotype block map for *SP110* with 10 SNPs. **b** Association of haplotype frequencies with TB risk in TB cases vs. LTBI individuals.

a

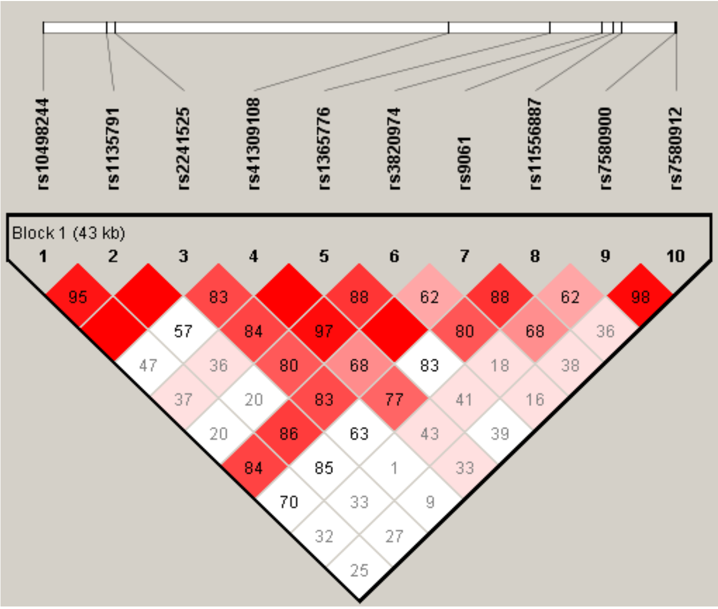

b

Haplotypes	Frequencies		Chi Square	OR (95%CI)	*P* value
	TB	Health			
ATGTACGCAA	0.287,	0.280	0.072	1.04 (0.80-1.34)	0.7885
ATATACGCAA	0.081,	0.094	0.588	0.85 (0.56-1.28)	0.4431
ATGTACGCGG	0.070,	0.059	0.576	1.20 (0.75-1.94)	0.4477
ATATACGCGG	0.061,	0.061	0	0.99 (0.61-1.63)	0.9937
GCGTACGCAA	0.047,	0.053	0.217	0.88 (0.52-1.51)	0.6412
ATGAAAACGG	0.051,	0.042	0.55	1.24 (0.71-2.16)	0.4581
ATGTGAGCAA	0.035,	0.045	0.742	0.77 (0.42-1.40)	0.389
ATGAAAGCAA	0.034,	0.045	0.889	0.75 (0.41-1.37)	0.3458
ATGAAAATGG	0.027,	0.047	2.988	0.58 (0.30-1.09)	0.0839
GCGTGAGCAA	0.041,	0.027	1.693	1.54 (0.80-2.98)	0.1932
ATGAAAGCGG	0.033,	0.025	0.706	1.35 (0.67-2.73)	0.4009
ATGTACGCGA	0.025,	0.027	0.026	0.94 (0.45-1.96)	0.8714
ATGAAAATAA	0.023,	0.025	0.061	0.91 (0.42-1.97)	0.8057
ATGTACACGA	0.024,	0.023	0.027	1.07 (0.49-2.30)	0.8702
ATGAAAATGA	0.023,	0.021	0.08	1.12 (0.51-2.49)	0.7774
GCGTACGCGG	0.024,	0.007	5.709	3.81 (1.18-12.31)	***0.0169***
ATGTGAGCGG	0.019,	0.009	1.695	2.00 (0.69-5.81)	0.1929

1 OR: odds ratio; CI: confidence interval; ORs are adjusted for gender. The significant ORs are shown in italic.

Figure S3: Association of *SP110* SNP haplotypes with TB risk in TB cases vs. healthy controls. **a** Haplotype block map for *SP110* with 10 SNPs. **b** Association of haplotype frequencies with TB risk in TB cases and healthy controls.

Table S1: Primers for genotype analysis for *SP110*

Gene	SNP	Primer	Sequence	Size (bp)	Allele 1 mass	Allele 2 mass
SP110	rs7580912	Forward	ACGTTGGATGGTCTCAAACCACAAACCACC	99		
		Reverse	ACGTTGGATGCCCCACCTTCTGTGATAATG			
		Extension	CTGGGCCTTCCAAACTC		G, 5353.5	A, 5433.4
SP110	rs7580900	Forward	ACGTTGGATGGTCTCAAACCACAAACCACC	99		
		Reverse	ACGTTGGATGCCCCACCTTCTGTGATAATG			
		Extension	TGTGATAATGAACATGCC		G, 5769.8	A, 5849.7
SP110	rs11556887	Forward	ACGTTGGATGATGAATGGCAGAGCAGAGAC	100		
		Reverse	ACGTTGGATGTGGGGTATGGAGGGAGCTT			
		Extension	TGAAGCCCCAACTGGCCTAG		C, 6334.2	T, 6414.1
SP110	rs9061	Forward	ACGTTGGATGCTCCTTCAGTGACCAATGAC	111		
		Reverse	ACGTTGGATGTGCACAGTGCTAGTGAGGAG			
		Extension	TACATCCAAAATGAATGCG		A, 6076	G, 6092
SP110	rs3820974	Forward	ACGTTGGATGTTTCTCCTCATTCTGGTCCC	98		
		Reverse	ACGTTGGATGTTGTCACTGGCCACTGAATG			
		Extension	ACTGAATGGAGGAAGAAAAAG		C, 6872.5	A, 6912.4
SP110	rs1365776	Forward	ACGTTGGATGAGATGTATCTGGTCAACTCC	102		
		Reverse	ACGTTGGATGCAGCTTCCTCTTGTACTCTC			
		Extension	TGTACTCTCATCTTACCTC		G, 5911.9	A, 5991.8
SP110	rs41309108	Forward	ACGTTGGATGTCCTGGGTCTCTGGGAGATT	106		
		Reverse	ACGTTGGATGAGTGCCATCAATGATCTCTG			

		Extension	TAGTAAATGCCCCTTGGGAAAC		T, 7014.6	A, 7070.5
SP110	rs2241525	Forward	ACGTTGGATGTCCCTAGATGAAG GACTCAC	98		
		Reverse	ACGTTGGATGTAGCTAGCAGGGT CCATTTC			
		Extension	TCCTGATCAATTACACACA		G, 5962.9	A, 6042.9
SP110	rs1135791	Forward	ACGTTGGATGGAAGGAAAAGGA AGGAACGC	103		
		Reverse	ACGTTGGATGAATACCTTCAGCAG CTCTCC			
		Extension	ACGGAATATACGTTGTGAAGGAA		C, 7407.9	T, 7487.8
SP110	rs10498244	Forward	ACGTTGGATGCAGACTCTCTGCTT GTTGTG	117		
		Reverse	ACGTTGGATGCAGAGTGGATATCC AAACCC			
		Extension	TGTGCCATTTGTCTTGC		A, 5429.6	G, 5445.6
